# An Overview of Lipid Droplets in Cancer and Cancer Stem Cells

**DOI:** 10.1155/2017/1656053

**Published:** 2017-08-13

**Authors:** L. Tirinato, F. Pagliari, T. Limongi, M. Marini, A. Falqui, J. Seco, P. Candeloro, C. Liberale, E. Di Fabrizio

**Affiliations:** ^1^German Cancer Research Center (DKFZ), Heidelberg, Baden-Württemberg, Germany; ^2^Physical Science and Engineering (PSE) Division, King Abdullah University of Science and Technology (KAUST), Thuwal, Saudi Arabia; ^3^Biological and Environmental Science and Engineering (BESE) Division, King Abdullah University of Science and Technology (KAUST), Thuwal, Saudi Arabia; ^4^Department of Applied Science and Technology (DISAT), Politecnico di Torino, Torino, Italy; ^5^BioNEM Lab, Department of Experimental and Clinical Medicine, University Magna Graecia of Catanzaro, Catanzaro, Italy

## Abstract

For decades, lipid droplets have been considered as the main cellular organelles involved in the fat storage, because of their lipid composition. However, in recent years, some new and totally unexpected roles have been discovered for them: (i) they are active sites for synthesis and storage of inflammatory mediators, and (ii) they are key players in cancer cells and tissues, especially in cancer stem cells. In this review, we summarize the main concepts related to the lipid droplet structure and function and their involvement in inflammatory and cancer processes.

## 1. Introduction

**Figure 1:**
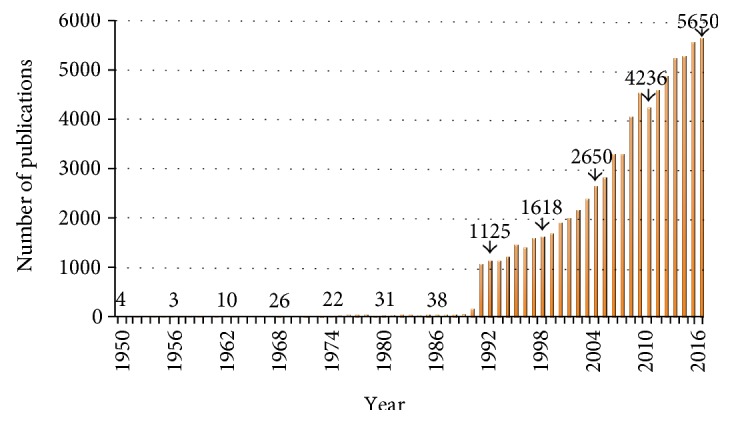
Growth of Web of Science-indexed publications, by year, using the key words “lipid droplets,” “lipid bodies,” “adiposomes,” or “oil bodies,” from 1950 to 2016.

Lipid droplets (LDs) have been considered for a long time as the fat storage compartment in cellular metabolic processes, and only recently have they drawn an increased attention by the scientific community. A quick search in the Web of Science database using any of the following search strings “lipid droplets,” “lipid bodies,” “adiposomes,” or “oil bodies” [1] yields more than 85,000 articles (Figure 1), almost all of them published in the last 20 years. In fact, LDs are now considered as dynamic and functional organelles not only responsible for fat storage but also involved in membrane biosynthesis, lipid metabolism, cell signaling, inflammation, and cancer [2–4].

In this review, we present an initial overview of LDs, indicating their lipid and protein composition and the major models of LD biogenesis. Then, we focus on the role of LDs in cancer and their presence in cancer stem cells (CSCs), highlighting a potential link between LDs and cancer stemness. In this regard, Raman spectroscopy can provide a new and powerful tool for the investigation and characterization of LDs in different living cellular systems.

The review does not address individual lipid signaling pathways and their interplay with glucose metabolism in cancer and other diseases, which can be found elsewhere.

## 2. LD Composition and Biogenesis

LDs are spherical organelles with size ranging from a few dozens of nanometers to hundreds of micrometers depending on cell type in which they are found. Depending on the tissue of origin, they contain variable ratios of neutral lipids, such as cholesteryl esters (CEs), retinyl esters, and triglycerides (TAGs) with saturated or unsaturated chains. Further, they are surrounded by a single layer of phospholipids, with phosphatidylcholine as the most abundant component, and various kinds of proteins [5–7]. Differences in size and amount of LDs, as well as in their lipid/protein composition, may reflect not only differences among cell types (intercellularly) but also differences between cellular metabolic states of a single cell type (intracellularly). In addition, *in vitro* LDs are also dependent on the culture conditions, while *in vivo* LDs are influenced by resting, fasting, or pathological status. LDs are found in almost all human cells, particularly in hepatocytes, enterocytes, and adipocytes [7].

Evidence shows that some protein components, present on LD surface, are derived from endoplasmic reticulum (ER) [8, 9]; in fact, the enzymes involved in TAG and CE synthesis reside on the ER membrane.

**Figure 2:**
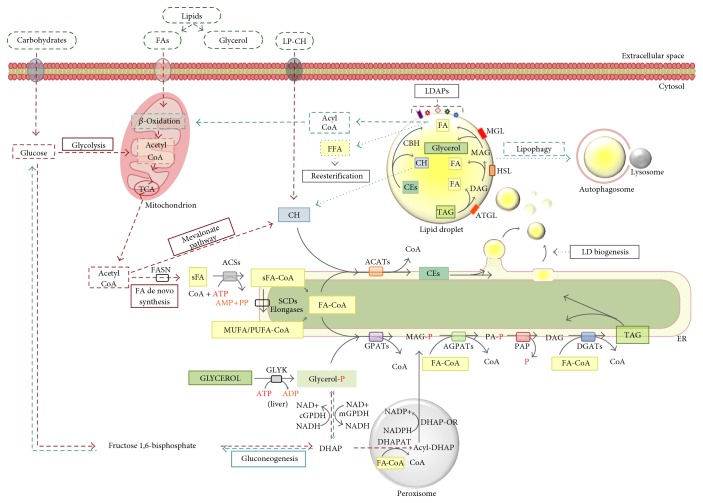
Schematic overview of the metabolic pathways required for the de novo synthesis of triacylglycerols and their lipolysis. Simplified representations of de novo FA synthesis and LD biogenesis are also included. FA-coA and MUFA/PUFA-coA are in general referred to as FA-CoA. AMP: adenosine monophosphate; ATP: adenosine triphosphate; ACATs: acyl-coA:cholesterol acyltransferases; ACS: acyl-coA synthetase; AGPATs: 1-acyl-glycerol-3-phosphate acyltransferases; ATGL: adipose tissue triacylglycerol lipase; CEH: cholesteryl ester hydrolase; CEs: cholesteryl esters; CoA: coenzyme A; DAG: diacylglycerol; DGAT: diacylglycerol acyltransferase; DHAP: dihydroxyacetone phosphate; DHAP-OR: dihydroxyacetone phosphate oxidoreductase; DHAPAT: dihydroxyacetone phosphate acyltransferase; ER: endoplasmic reticulum; FA: fatty acid; FA-CoA: fatty acyl-coenzyme A; FFA: free fatty acid; sFA: saturated FA; FASN: fatty acid synthase; GLYK: glycerol kinase; GPATs: glycerol-3-phosphate acyltransferases; cGPDH: cytosolic glycerol-3-phosphate dehydrogenase; mGPDH: mitochondrial glycerol-3-phosphate dehydrogenase; HSL: hormone-sensitive lipase; LDAPs: lipid droplet-associated proteins; LP-CH: lipoprotein involved in transporting cholesterol; MAG: 1-acylglycerols; MGL: monoacylglycerol lipase; MUFA: monounsaturated FA; NAD: nicotinamide adenine dinucleotide; NADH: reduced nicotinamide adenine dinucleotide; NADP: nicotinamide adenine dinucleotide phosphate; NADPH: reduced nicotinamide adenine dinucleotide phosphate; P: phosphate; PA-P: phosphatidic acid; PAP: phosphatidic acid phosphatase; PUFA: polyunsaturated FA; SCDs: stearoyl-CoA desaturases; TAG: triacylglycerol; TCA: tricarboxylic acid cycle.

Ultrastructural analysis of LDs shows that they are often found in intimate contact with both the (i) mitochondria, where the *β*-oxidation of fatty acids takes place, and with (ii) ER cisternae, suggesting a strong relationship between these organelles [10] (Figure 2).

Based on the prevailing budding model for LD formation, newly synthesized neutral lipids accumulate inside the ER membrane bilayers, from which the cytoplasmic leaflet buds off taking phospholipids and ER membrane proteins. Lipids are channeled into this nascent LD, which is initially tethered to ER. The new LD is then released into the cytosol [11]. It has been proposed that Arf1/COPI complexes may trigger the formation of bridges between the ER and the nascent LD [12]. In an alternative model, neutral lipids accumulate inside the ER bilayer, forming an oil lens, which is subsequently excised [13]. Once released into the cytoplasm, LDs tend to increase their volume either by localized lipid synthesis [9], transport of lipids to LDs [14], or by fusion with other LDs (Figure 2) [15, 16].

Furthermore, while LDs are generally considered to be located in the cell cytoplasm, very recently, Ohsaki et al. [17] have convincingly demonstrated the presence of LDs inside the nuclei of several human and mammalian cell lines by using confocal and electron microscopy. The authors also investigated the molecular basis for nuclear LD formation. In light of these considerations, the suggested model of LDs as unique subcellular domains (niches) proposed by Welte [4] offers several intriguing hints.

## 3. LD Synthesis and Catabolism

Due to the potential toxicity of fatty acids (FA) to cells, surpluses of nonesterified FAs and cholesterol (CH) are stored within the LDs as neutral inert molecules, such as TAGs or sterol esters. The TAGs are made of three FA chains bound to a glycerol backbone. TAGs are synthesized by a complex pathway (Figure 2()), which initially requires the following: (i) the activation of saturated and/or unsaturated FAs to fatty acyl-coenzyme A (FA-CoA) esters by an acyl-CoA synthetase (ACS) activity and (ii) the phosphorylation of glycerol by glycerol kinase (GLYK), which is found in the liver, or the cytosolic synthesis of glycerol-3-phosphate from dihydroxyacetone phosphate (DHAP) produced during glycolysis by glycerol-3-phosphate dehydrogenase (GPDH) enzyme, which mainly occurs in the liver and adipose tissues. In the adipose tissue and, at lesser extent, in other tissues, glycerol-3-phosphate is also derived from peroxisomal conversion of the DHAP in a pathway involving a first acylation catalyzed by the dihydroxyacetone phosphate acyltransferase (DHAPAT), followed by a reduction by 1-acyl-dihydroxyacetone phosphate oxidoreductase (DHAP-OR) [18].

Once formed, FA-CoA is used for the first acylation of glycerol-3-phosphate via glycerol-3-phosphate acyltransferase (GPAT) enzymes, which produces 1-acylglycerol-3-phosphate (MAG-P) (Figure 2()). Then, 1-acyl-glycerol-3-phosphate acyltransferase (AGPAT) catalyzes the second acylation converting MAG-P in 1,2-diacylglycerol phosphate (phosphatidic acid (PA-P)), which in turn is dephosphorylated to 1,2-diacylglycerol (DAG) by phosphatidic acid phosphatase (PAP or lipin). PAP is a cytosolic Mg^2+^-dependent enzyme able to transiently localize to the ER membrane for catalyzing the phosphatase reaction [19, 20]. Lastly, acyl-CoA:diacylglycerol acyltransferase 1 and 2 (DGAT1 and DGAT2) enzymes catalyze the third esterification of DAG into TAG (Figure 2()) [19].

Another major component of LDs is represented by CE, which derives from the esterification reaction between FA-CoA and CH performed by acyl-CoA:cholesterol acyltransferase 1 and 2 (ACAT1 and ACAT2). ACAT1 is expressed in all tissues, while ACAT2 is mainly present in the intestine and liver [21]. CH is used for membrane synthesis and repair and, in steroidogenic cells, as a precursor for steroid hormone synthesis [22].

Newly synthesized TAGs and CEs are then stored in LDs or, in the liver, secreted in the blood in the form of very-low-density lipoproteins (VLDL) to be delivered to other tissues.

On the other hand, TAGs can be mobilized from within LDs to produce energy (Figure 2). This can occur through two different processes: via degradation by cytoplasmic triglyceride lipases (CTLs) recruited to the LDs, such as adipose tissue triacylglycerol lipase (ATGL) and hormone-sensitive lipase (HSL), or by lysosomal lipase following autophagic pathways. In the former, ATGL bound on LD surface hydrolyzes TAGs to DAGs, and HSL, after translocation from cytosol to LDs, converts DAGs into 1-acylglycerols (MAGs), and finally monoacylglycerol lipase (MGL) hydrolyzes MAGs into free FAs (FFAs) and glycerol. FFAs may be delivered to the mitochondria for the *β*-oxidation, in order to obtain energy, or used as substrates for reesterification, for membrane synthesis, or as signaling molecules [20, 23]. In the latter, LDs are incorporated in autophagosomes, which fuse with lysosomes forming autolysosomes, in a process named “lipophagy” [24]. Lipophagy is supposed to be the principal mechanism of LD catabolism in hepatocytes, where ATGL and HSL are less expressed. Lysosomes contain the lysosomal acid lipase (LAL) that hydrolyzes TAGs and CEs and proteases that degrade proteins, but the biochemical processes and molecular mechanisms underlying are poorly known (Figure 2) [4, 7, 20].

## 4. Protein Composition of LDs

The proteins associated with LDs coat the surface of membrane monolayer and participate in LD formation, growth, trafficking, and catabolism. Different proteins have been identified by proteomic analysis, mostly classified into three groups: (1) structural proteins, such as the members of the PAT (perilipin-ADRP-TIP47) family and the cell death-inducing DFF45-like effector (CIDE) family; (2) membrane-trafficking proteins, including but not limited to Rab10, Rab18, Rab32, and Arf1 proteins and soluble NSF attachment protein receptors (SNAREs); and (3) enzymes implicated in lipid synthesis, such as DGAT2, and catabolism, such as ATGL and HSL [8, 25].

LD-associated proteins can be cytosol-derived proteins or ER-derived proteins [26]. Their regulation is finely controlled and responds to physiological conditions, like fasting and feeding and hormones. Further, their expression varies depending on cell and tissue types. Different PLIN combinations on the LD surface would confer LD tissue specificity [27, 28]. Interestingly, Hsieh et al. showed that LD composition and localization can vary at a single cell level. In particular, FA-enriched LDs localize preferentially to the cell periphery, while CE-enriched LDs to central regions. Besides, in various cell types, different exogenous lipid stimuli exert differential effects on perilipin coating and differential targeting of perilipins to different classes of LDs [29].

**Figure 3:**
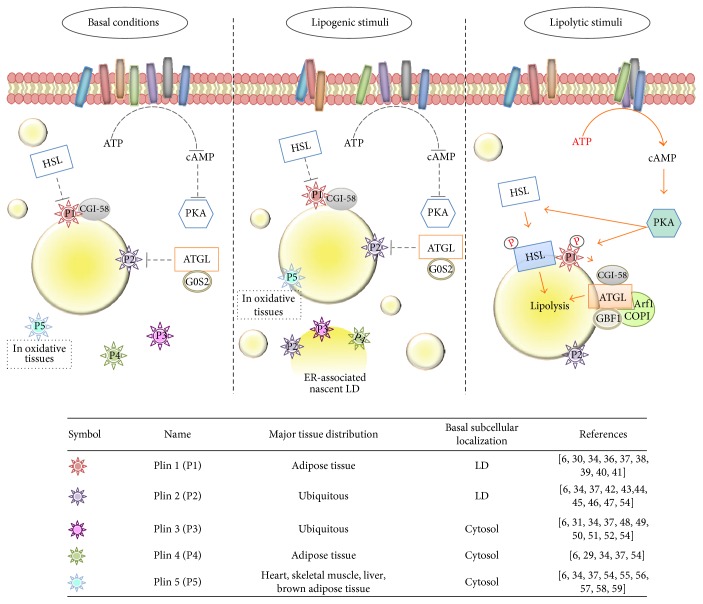
Schematic model representing the mechanisms of PLIN1 and PLIN2 action and PLIN3, PLIN4, and PLIN5 localization. In basal conditions (left panel), PLIN1 forms a complex with CGI-58, while ATGL association with G0S2 impairs the enzyme activity. PLIN1 and PLIN 2 block the LD access to lipases, and only a low rate of lipolysis takes place. Under lipogenic stimuli or FA surplus (central panel), PLIN2, PLIN3, and PLIN4 localize on nascent LDs at ER. Lipolytic stimuli (right panel), such as *β*-adrenergic stimulation, activate PKA via increased levels of cAMP. PKA phosphorylates PLIN1 and HSL, inducing HSL translocation to LD surface and release of CGI-58. This latter can form a complex with ATGL, whose activity also requires the binding with GBF1 factor and with Arf/COPI complex resulting in activated lipolysis. GBF1: Golgi brefeldin A resistance guanine nucleotide exchange factor 1; G052: 33mer gliadin peptide; CGI-58: comparative gene identification-58; P1: perilipin 1; P2: perilipin 2; P3: perilipin 3; P4: perilipin 4; P5: perilipin 5; PKA: protein kinase A; Arf1/COPI: ADP ribosylation factor/coat protein complex.

The PAT family includes five members: perilipin 1 (PLIN1) [30], perilipin 2 (PLIN2/ADRP/adipophilin) [6], perilipin 3 (PLIN3/TIP47) [31], perilipin 4 (PLIN4/S3–12) [32], and perilipin 5 (PLIN5/OXPAT) [33, 34]. It can be also distinguished in constitutively LD-localized proteins (PLIN1 and PLIN2) or exchangeable LD-localized proteins following lipogenic or lipolytic stimuli (TIP47, S3-12, and OXPAT) (Figure 3) [35].

PLIN1 represents the most abundant structural protein on the LDs and is highly and stably expressed in mature adipocytes of white and brown adipose tissue and at lesser levels in macrophages [34, 36, 37].

A recent study showed that the lack of PLIN1 correlates with the attenuation of the nuclear SREBP-1 expression, finally resulting in decreased LD formation [38]. The proposed acting mechanism is the control of the interaction/accessibility of internal stored lipids and external cytosolic lipases that results in the regulation of lipolysis. In fact, under basal conditions, PLIN1 hinders the relocation of cytosolic lipase HSL to LDs and blocks ATGL activity by binding with the cofactor perilipin-associated comparative gene identification-58 (CGI-58) requested for ATGL activation (Figure 3) [39]. On the other hand, under conditions of energy requirements, the active protein kinase A (PKA) phosphorylates PLIN1 and HSL, fostering translocation and binding of HSL on the LD surface [40, 41]. In addition, CGI-58 is released, which activates ATGL and localizes it to the LDs, thus stimulating TGA lipolysis [40, 41] (Figure 3). These observations point out to an important role played by PLIN1 in LD formation and TGA metabolism [37].

PLIN2 is a ubiquitous mainly associated LD protein, particularly abundant in the liver [42], whose expression is positively correlated with TAG levels and LD formation [43]. During adipogenic differentiation of 3T3-L1 preadipocytes, PLIN2 is replaced by PLIN1 [44]. It has been suggested that, by hampering the association of ATGL with LDs, PLIN2 hinders lipolytic pathways, which results in increased TAG levels and in the accumulation of LDs (Figure 3) [45–47].

Similarly to PLIN 2, PLIN3 is also distributed in many tissues, but, unlike PLIN1 and PLIN2, it localizes to the cytosol [31, 48, 49]. In the presence of a FA surplus, it translocates from cytoplasm to nascent LDs stimulating TAG biosynthesis and storage (Figure 3). This was suggested by the knockdown of PLIN3 in THP-1 cells, in which TAG level decreased, while they accumulated in the presence of full-length PLIN3 [50]. Additionally, it displays apolipoprotein-like properties, the ability to bind to DAG-rich ER membrane sites during LD formation and to LDs during TGA mobilization [51, 52].

PLIN4 is selectively expressed in adipocytes and to a lesser degree in the skeletal and cardiac muscles [53, 54]. Like PLIN1, PLIN4 appears in the last stages of the adipocyte differentiation, but differently, it is localized in the cytosol under basal conditions. In the presence of adipogenic stimuli, PLIN4 coats nascent LDs together with PLIN3 and PLIN2 [37, 54] and it seems to preferentially target CE-enriched LDs [29].

PLIN5 expression is specific for oxidizing tissues, such as heart, liver, and brown adipose tissue, and recently, it has been also discovered in hepatic and pancreatic cells [55, 56]. It is a cytosolic perilipin able to translocate to LDs following lipogenic stimulation, and PLIN5-associated LDs are also found in close proximity with mitochondria (where the FA *β*-oxidation occurs) in muscle cells [57]. Studies of the hearts of PLIN5 knockout mice and of mouse hearts overexpressing PLIN5 are consistent with a PLIN5 protective role of LD storages against lipase activity and presumptively against the toxicity induced by excessive FA oxidation [58, 59].

Altogether, these observations suggest that PLIN1, PLIN2, and PLIN5 could regulate lipid metabolism by interacting with lipolytic enzymes or, more probably, by impairing the access of lipases to LDs, while PLIN3 and PLIN4 would be involved in the control of the intracellular neutral lipid packaging and trafficking [54].

The CIDE is another important family of LD coat proteins, including CIDEA [60], CIDEB, and CIDEC (or FSP27). CIDEA and CIDEB have been shown to reside on LDs and on the ER of brown adipose tissue and in the liver, respectively, while CIDEC in white and brown adipose tissues, but not in normal liver tissues [61]. Recent findings reveal that CIDE proteins are localized at the contact site between different LDs, where they probably mediate lipid transfer, LD fusion, and growth [62, 63]. It has been proposed that the interaction between FSP27 and PLIN1 would assist FA exchange through a channeling pore between LDs and would be crucial to protect from lipotoxicity through the accumulation of TAGs and the formation of large LDs [30, 64].

Among membrane-trafficking proteins, a variety of GTPase of the Rab family, Arf1, and SNARE proteins, as well as caveolins and cavins, have been also described to target to LDs [1, 6, 65–68]. These proteins seem to be entailed in trafficking and sequestration of proteins to LDs and in lipid mobilization from LDs with the aim at delivering them to different cellular compartments, at regulating their levels, and/or at hampering their binding with target partners [69, 70]. In this context, Rab GTPases are the most abundant class of proteins associated to LDs, even though, only for some of them, such as Rab1, Rab5, Rab10, Rab18, and Rab32, a functional interaction with LDs has been reported [68, 71]. Recent evidence also suggests a crucial role of SNARE proteins in the fusion of LDs and of Arf1/COP-I complex, which would allow ATGL to target LDs by inducing the dissociation of PLIN2 (Figure 3) [25, 68].

Finally, concerning the enzymatic proteins involved in lipogenesis and lipolysis, ATGL and HSL are the most studied members of the lipase family activated under lipolytic stimuli. In particular, HSL is a cytosolic enzyme, while ATGL is a LD-associated protein [72]. As mentioned before, after the activation by the PKA phosphorylation, HSL translocates from cytosol to LDs where it is responsible for the hydrolysis of DAGs into MAGs (Figure 3) [20, 41].

ATGL accessibility to TGA stored inside LDs appears mediate by Arf1/COPI complex, which would transport ATGL to LDs from the ER [12]. Moreover, a recent finding showed that Golgi brefeldin A resistance factor 1 (GBF1), an exchange/activator factor of Arf1, also intervenes in the translocation of ATGL from ER membrane to LD surface [73], though the mechanisms underlying are still poorly understood. Additionally, cofactor CGI-58 and the peptide G0G1 switch protein 2 (G0S2) appear to be important elements in the activation of ATGL or in its inhibition, respectively, as well as in the ATGL transfer from ER to LDs (Figure 3) [39, 74].

Finally, DGAT enzymes, especially DGAT2, would be present on LDs to recycle hydrolyzed lipids and expanding their core under FA surplus [75].

Therefore, LD-associated proteins are linked to both LD formation and interplay with other cytoplasmic organelles through complex pathways and, to date, the mechanisms involved in targeting and recruitment of the proteins from cytosol and ER onto LD surface, as well as the protein-specific contribution to LD homeostasis, remain to be elucidated. For a more exhaustive description of LD-associated proteins, we refer the readers to more detailed reviews [26, 71, 76].

## 5. Lipid Droplets and Cancer

While LD's responsibility in obesity and related diseases has been extensively investigated [1], only in recent years has their implication in cancer attracted the interest of scientists. As far back as 1963, Aboumrad et al. described a class of mammary carcinoma characterized by a high number of stainable lipid vesicles in the cytoplasm [77]. Since that time, the aforementioned lipid particles were considered as a not specific degenerative change related to that neoplasm. In 1973, Ramos et al. clinically and morphologically characterized 13 patients with lipid-containing mammary carcinoma and categorized these tumors as a distinctive clinic-pathologic variety with a more aggressive behavior [78]. Since then, lipid-rich carcinoma continued to be reported in human and animal studies [79, 80]. Nevertheless, the roles of lipids in cancer development were not clearly understood and a widely accepted classification of lipid-rich tumors as a clinically distinctive form of carcinoma was lacking [81].

Nowadays, there is a general consensus that cancer cells display metabolic reprogramming compared to healthy cells, related not only to mechanisms of ATP synthesis through glycolysis (Warburg effect) [82] but also to de novo lipid synthesis, with fatty acid synthase (FASN) and sterol regulatory element-binding protein (SREBP) family as key players in many human cancers [83–89]. Under physiological conditions, normal cells tend to maintain lipid levels under control, by regulating uptake, synthesis, and mobilization from internal storages. By contrast, tumor cells are able to uptake larger amount of lipids, as well as to enhance lipogenesis and CH production, and to increase FA *β*-oxidation [90, 91]. How these changes occur and which molecular pathways are involved remains poorly understood. Many healthy adult mammalian tissues preferentially use exogenous FAs for their needs maintaining low levels of FASN, the enzyme catalyzing the last step of FA synthesis. Conversely, increased cholesterol biosynthesis and high rate of synthesis and oxidation of endogenous FAs have been reported in cancers from different tissues and they have been correlated with unfavorable outcomes [83, 92, 93]. In colorectal (CR) cancer, FASN hyperactivation promotes LD accumulation and endogenous FA *β*-oxidation, during metabolic stress [86]. Moreover, the role of the lipogenic ACS enzymes is also intensively investigated for its involvement in tumor cell proliferation and tumorigenicity [94].

However, both de novo lipogenesis and upregulation of lipolysis from intracellular storages translate in increased FA availability for transforming cells and they seem accompanying the pathogenesis of cancer disease. By proteomic analysis, Nomura and coworkers demonstrated that different aggressive types of cancers displayed a high expression and activity of the lipolytic MGL enzyme, compared to not aggressive counterparts. This increased activity correlated to higher free FA levels liberated from lipid stores and promoted tumor aggressiveness, most likely due to the modulation of protumorigenic lipid messengers, such as LPA and PGE2 [95]. In fact, lipids in cancer are most likely required not only for sustaining rapid proliferation rate and a high energy consumption [96] but also for stimulating signaling pathways involved in cell survival, angiogenesis, and metastatic processes by acting as second messengers [86, 95, 97]. Bioactive lipids, such as phosphatidylinositol, phosphatidylserine, or LPA, are recognized as important signaling factors able to modulate proliferative and survival pathways, in particular the PI3K/AKT, Ras, or Wnt pathways [98].

Lipogenesis in cancer cells could have a role in making cells less sensitive to lipid peroxidation by increasing the saturation levels of fatty acyl chains of membrane phospholipids thus altering their properties [99]. This in turn translates, on one hand, into modulation of effectors and pathways inside cells, such as ER stress responses [100], and, on the other hand, into regulation of the crosstalk between tumor cells and stroma, which may be crucial for the progression of the transformed phenotype and for drug resistance [83, 101, 102]. It has been demonstrated that ovarian cancer cell growth depends on lipids derived from adipocytes grown in coculture experiments and that such transfer induces FA *β*-oxidation [103].

In order to avoid lipotoxicity due to an excess of lipids in the cytoplasm, lipid and CH storage is ensured by LD formation. Cancer cells accumulate a larger number of LDs in their cytoplasm when compared to normal cells [104]. For example, breast and prostate cancers are associated to high LD content. In breast cancer, this phenomenon has been correlated with the presence of estrogen/progesterone receptors, which are well-known modulators of cell signaling pathways involved in cell cycle, angiogenesis, and metastasis but also known to trigger lipogenic pathways, including the FASN signaling [105, 106]. Treatment of breast cancer cells with hormone medroxyprogesterone acetate results in an increasing number and size of LDs, which are preferentially enriched in saturated lipids, as revealed by Raman spectroscopy [107]. However, estrogen receptor-negative breast cancer cell lines also show high LD accumulation associated with a higher lipid uptake. This may probably confer an energetic advantage and favor the development of a more aggressive phenotype [108]. Also, breast lipid-rich carcinomas are usually, but not always [109], negative for the expression of estrogen receptors [110], thus implying more complex levels of regulation/stimulation of lipid synthesis and storage.

LD role in cancer is only beginning to be explored, and recent evidence suggests that higher levels of LDs are associated with higher tumor aggressiveness [111] and chemotherapy resistance [112]. Moreover, some studies report the accumulation of proteins involved in tumorigenesis, such as phosphatidylinositol-4,5-bisphosphate 3-kinase (PI3K), extracellular signal-regulated kinase 1 and 2 (ERK1 and ERK2), and caveolins in LDs of different cancer cells [113–116]. LDs also appear to be associated with inflammatory responses, which can result in initiation and progression of neoplastic processes [117, 118]. In fact, LDs have been shown to be specialized sites involved in compartmentalization and amplification of inflammatory mediators, such as arachidonic acid, eicosanoids, and enzymes required for their synthesis [115, 119]. Recently, a correlation between prostaglandin E2 (PGE2) synthesis and increased LD levels in inflamed colonic tissues has been reported [120]. Further, in an experimental work by Accioly et al. [121], the authors showed that human colon adenocarcinoma cell lines and colon cancer biopsies from patients present a huge increase of LDs when compared with those from their healthy counterpart. Moreover, they have also found that LDs contain COX-2 and are structurally distinct cytoplasmic sites for PGE2 production in an adenocarcinoma cell line [121]. Noteworthy, PGE2 is the most abundant prostaglandin found in several human malignancies such as colon, lung, breast, and brain [122–125] and some evidence underlies its crucial role in promoting tumor growth [126]. It should be also noticed that PGE2 not only promotes tumor growth not exclusively in a paracrine way but also regulates the interaction between tumor cells and the surrounding stromal cells [101]. This mechanism would allow tumor cells to escape the immune system attack, thus promoting immunosuppression [127]. PGE2 seems also to cause myeloid-derived suppressor cell activation through an exosome-dependent transport [101, 128, 129].

Penrose et al., have provided recent evidence of a link between LD increase and epidermal growth factor receptor (EGFR) in CR cancer. In their work, EGFR activation stimulates de novo lipogenesis with consequent accumulation of LDs expressing increased levels of PLIN2 protein in various CR cancer cells. These effects were positively mediated by the EGFR-induced activation of the PI3K/mTOR pathway and of PGE2 synthesis and negatively mediated by the inactivation (loss) of FOXO/SIRT6 tumor suppressor factors. However, the degree of these effects varied depending on cell line [43]. PI3K is an upstream regulator of FOXO activity; thus, this work supports a potential regulative role of this axis in modulating LD content in cancer cells and suggests a novel molecular link between LDs and tumor growth. It should be noticed that LD accumulation relies on cancer cell metabolic status and that different genetic profiles inside a cell population can generate heterogeneity in terms of LD content (LD^High^ and LD^Low^ cells), defining different adaptive responses [130, 131].

In another recent paper published by Zirath et al. [132], the accumulation of LDs in neuroblastoma cells has been also observed following the inhibition of the transcriptional regulator, MYCN, owing to alterations of the mitochondrial respiratory chain. Moreover, cancer cell survival by upregulation of autophagy has been demonstrated in some advanced tumors [133] and, in this respect, LDs could represent a source of membrane lipids and energy in autophagosome biogenesis [134, 135], thus suggesting a wider scenario for LDs in tumorigenesis.

## 6. Cancer Stem Cells

The cancer stem cell hypothesis proposes that inside a tumor mass, a subset of cells with stem-like features and named cancer stem cells (CSCs) [136] can exist with the ability to self-renew and, at the same time, to generate heterogeneous differentiated cancer cells that make up the tumor [137]. CSCs sustain tumor growth, have the ability to spread into other organs, and show resistance to conventional therapies [138–140]. Moreover, when transplanted in immunocompromised mice, CSCs generate new tumors, which is consistent with the concept of heterogeneity of the original tumor (i.e., a mixture of stem, progenitor, and mature cells) [141–143]. This hypothesis has led to the view that cancer is a hierarchically organized structure, with CSCs responsible for tumor development, progression, and maintenance, as well as for heterogeneity [142, 144]. In this perspective, the contribution of microenvironment/niche cannot be overlooked. Tumor microenvironment consists of a cellular component (muscle, immune, endothelial, and stroma cells) and a biochemical component (growth factors and cytokines), spatially and temporarily orchestrated, which are believed to provide CSCs with altered stimuli able to influence cell neoplastic growth, functions, and metabolism [145]. CSCs have been identified in several types of cancers, such as myeloid leukemia [138, 141], breast [146], prostate [147], colorectal [148–150], lung [151], liver [139], melanoma [152, 153], and glioblastoma [154] cancers by using different sets of makers and assays, but not without controversy and limits [155]. For example, in CR cancer, CSC populations have been identified on the basis of the markers Lgr5 [156, 157], CD133 [148], BMI1 [158], Dclk1 [159], CD44 [150], and ALDH-1 [160]. In breast cancer, CSCs are characterized by high CD44 and low CD24 marker expression [146], while CD133 and CD44 are used for glioblastoma CSCs [154, 161].

Whether CSCs derive from genetically dysregulated stem cells (SCs), which lose the normal mechanisms of growth, differentiation, and apoptosis control, is still under debate. As a matter of fact, CSCs exhibit some similarities with SCs, including self-renewal and multipotency. Nevertheless, some evidence suggests the possibility that more differentiated cancer cells might also revert to a stem-like status as a result of a dedifferentiation process driven by genetic alterations, thus making all tumor cells stochastically able to have a tumor-initiating potential [143, 162]. Indeed, both hierarchical and stochastic models could favor tumor heterogeneity and contribute to cancer development, without the need for a reciprocal exclusion; cancer cell plasticity, the effects exerted by different and dynamic microenvironments, and the stage of cancer development could reconcile both models [163].

In the particular case of CR cancer, it has been recently showed that CSCs derive from normal SCs, following the activation of specific pathways, mainly Wnt/*β*-catenin [157]. On the other hand, more differentiated cells showing an overactivation of Wnt signaling, induced by an enhancement of NF-kB or by niche-secreted factors, can also dedifferentiate towards a more primitive stage and generate tumors [164, 165].

Compared to tumor bulk, CSCs show metabolic alterations, which are most likely essential to sustain their stem-like phenotype and vary depending on cancer type [166, 167]. For example, lower mitochondrial respiration and higher glycolytic rates have been observed in a model of osteosarcoma [168]. On the contrary, mitochondrial energy production by oxidative respiration rather than glycolysis has been reported in leukemia, glioblastoma, and pancreatic CSCs [169–171].

De novo FA synthesis is upregulated in CSCs due to the activation of intrinsic lipid pathways whose regulation is still poorly understood. Together with the overexpression of FASN even in CSCs [172], acyl-CoA synthetase ACSVL3 expression is increased in CSCs of glioblastoma neurospheres and its regulation positively depends on the activation of the oncogenic receptor tyrosine kinase pathway, thus correlating the altered FA metabolism with CSC maintenance and tumorigenesis [94].

**Figure 4:**
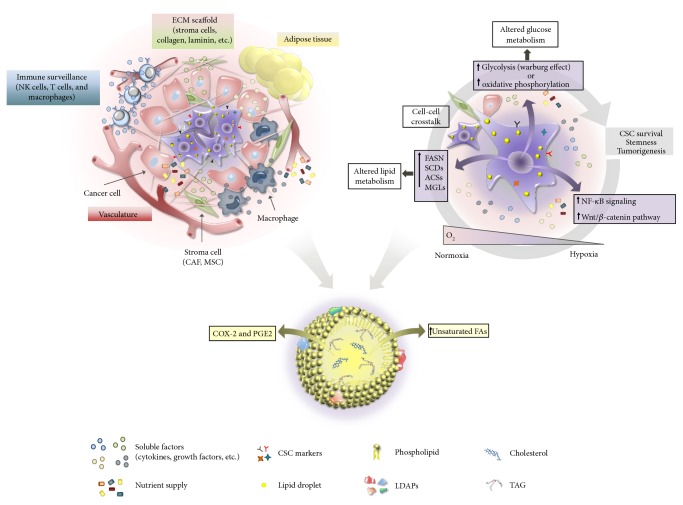
The schematic model depicts the hypothetic CSC niche (on the left) in human tumors. The main elements are summarized: (i) the cellular components, such as CSCs, cancer cells, adipocytes, immune cells, and stroma cells [e.g., cancer-associated fibroblasts (CAFs) and mesenchymal stem cells (MSCs)]; (ii) ECM components: collagen, laminin, and so forth; and (iii) soluble factors (growth factors, proinflammatory cytokines, chemokines, exosomes, etc.) release from different cell types and nutrient supply from vasculature. A CSC (on the right) shows higher amount of LDs compared with other cells. Within tumor bulk, hypoxia develops due to limited vascularization. A continuous interplay among different factors inside the niche, including hypoxic/normoxic conditions, nutrient supply availability, the release of soluble factors, and cell‐cell interactions contributes to determine the CSC properties and influences their metabolic plasticity, as reviewed in [143, 163, 166, 174]. A single cancerous LD (bottom) is also schematically represented, based on [121, 178].

Different conditions in the cancer niche, such as hypoxia/normoxia and nutrient supply, as well as levels of growth factors and cytokines may also act as modulators of CSC responses [143] even in terms of accumulation of LDs, in a continuous interplay inside the niche [173, 174] (Figure 4()). In this context, bioactive lipids and exogenous/endogenous FA availability may exhibit an important role in determining CSC behavior. Indeed, it has been shown that aggressive tumors often are surrounded by adipose tissue and home to adipocyte-rich metastatic sites [103, 173]. Variations in microenvironmental cues, CSC metabolic ability to adapt to different conditions, and the cellular heterogeneity inside the same cancer subtypes could explain the differences in CSC metabolic reprogramming reported in literature [164].

Therefore, exploiting metabolically altered pathways of CSCs represents an intriguing and at same time challenging task, which might allow to identify the key factors involved in CSC tumorigenicity. This would further help in developing novel therapeutic approaches specifically targeting this cellular subset.

## 7. Lipid Droplets in Cancer Stem Cells

Until recently, few studies have focused on potential correlations between lipid metabolism and stemness properties in CSCs. However, recently, there has been a growing interest in investigating the lipid metabolic profile in CSCs and evidence is accumulating on the key role of lipid molecules and consequently LDs, in CSC tumorigenicity.

Kashuba et al., in 2008, reported that overexpression of the mitochondrial ribosomal protein S18-2 alone led to immortalization of primary rat embryonic fibroblast inducing them to express stem cell traits [175]. Later, her group showed that the S18-2-immortalized cells underwent cell transformation and gave rise to tumors in SCID mice [176]. These cells showed induction of stem cell maintenance markers, such as Sox2 and Oct4, and the activation of some cellular pathways, such as cell proliferation, oxidative phosphorylation, and cellular respiration. Importantly, the most tumorigenic S18-2 clones had the largest amount of LDs when compared to the other clones, suggesting that the LD expression is functionally linked with increased cancer metabolism, stemness, and tumorigenicity [176].

As in more differentiated breast cancer cells, FASN expression appears upregulated in breast CSCs [177], while in ovarian ALDH^+^/CD133^+^ CSCs (OCSCs), a higher grade of unsaturated lipids has been found inside LDs, compared to non CSCs, by using Raman microspectroscopy and mass spectrometry [178]. This evidence has been associated to a higher expression of stearoyl-CoA desaturase-1 (SCD1), which catalyzes the synthesis of monounsaturated FAs. Thus, the high SCD1 levels and abundance of LDs enriched in unsaturated FAs would represent an altered metabolic feature of OCSCs and would have a functional role in the stemness maintenance, both *in vitro* and *in vivo*. The authors also provide evidence that the SCD1 activity and the nuclear factor NF-kB (NF-kB) pathway are regulated by a positive feedback loop [178]. However, the molecular mechanisms underlying this interaction are unknown.

It has been established that the hyperactivation of NF-kB signaling induces the expression of stemness-associated genes and of inflammatory genes in CSCs, thus suggesting a link between inflammation and tumorigenesis [179]. Recent observations pointed out to saturated FAs as potential factors stimulating inflammatory responses [180], with NF-kB acting as a main player. Moreover, among other factors, NF-kB activation is also mediated by the Toll-like receptor (TLR) family, expressed in different types of cancer [181, 182]. Interestingly, a recent paper demonstrated the ability of saturated FAs to activate TLR-2 and TLR-4 signaling pathways [183]. TLRs trigger inflammatory responses through the activation of transcription factors, including NF-kB, which can result in promoting cancer cell proliferation, invasion, and tumorigenesis [184]. For example, it has been shown that the TLRs/NF-kB pathway supports ovarian CSC self-renewal [185]. In light of these results, CSCs, LDs, and NF-kB signaling might be much more tightly connected than so far investigated.

**Figure 5:**
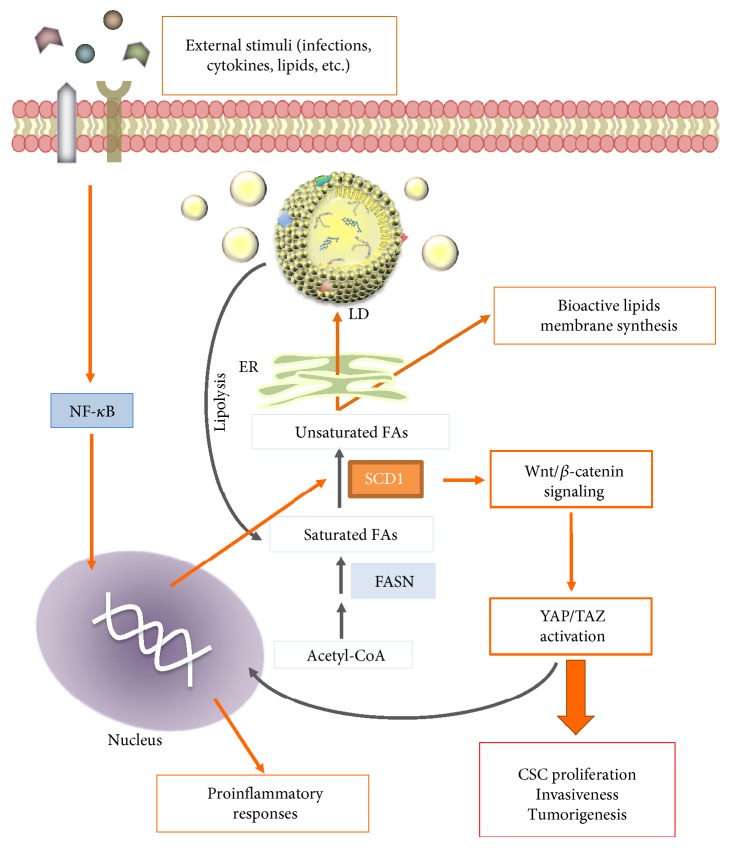
Hypothetical model of NF-kB/SCD1 pathway based on [177, 185]. Activated NF-kB pathway stimulates SCD1 upregulation, which could modulate several pathways (such as Wnt/*β*-catenin and YAP/TAZ signalings) resulting in CSC proliferation, cancer invasiveness, and tumorigenesis.

Notably, in Li et al.'s work, the upregulation of SCD1 in CSCs is associated with greater tumorigenicity and poor prognosis [178]. In a recently published paper, Noto et al. have shown that lung cancer stem cell spheroids display an increased amount of unsaturated FAs dependent upon the SCD1 activity. This latter induces the activation of Wnt/*β*-catenin signaling, and this axis, in turn, regulates the nuclear localization (activation) of YAP/TAZ, effectors involved in the Hippo pathway (Figure 5). Such a correlation was also associated with poor prognosis in test samples of human lung adenocarcinoma [186]. Actually, YAP/TAZ are inducers of stem cell proliferation and survival and, in several cancers, their expression sustains tumor growth and invasion [187, 188]. Also, YAP/TAZ activity seems to be modulated by metabolic pathways, including glycolysis and mevalonate pathway [189]. Thus, Noto et al.'s study provides evidence that the expression of SCD1 enzyme, from which at least in part depends a specific lipid composition of LDs, promotes cancer stemness, and describes a possible link between dysregulated lipid metabolism and YAP/TAZ oncogenic activity.

The LD accumulation has been also observed in circulating tumor cells (CTCs). Lipid-rich CTCs were detected in the peripheral blood of patients with metastatic prostate or lung cancers by using Raman spectroscopy. This supports the idea that intracellular lipids could be involved in cancer aggressiveness and that LDs could be used as a potential biomarker [81, 190].

Recently, a paper published by our group [131] demonstrated a higher accumulation of LDs in different patient-derived CR-CSCs compared to their nonstem counterparts. By measurements performed with different techniques, including Raman microspectroscopy, it has been shown that the traditional CR-CSC markers (CD133 and Wnt/*β*-catenin pathway activity) directly correlate with the cell fraction having the highest LD content (CR-CSC LD^High^). Furthermore, an *in vivo* test demonstrated that most of the tumorigenic potential is restricted to the CR-CSC LD^High^ subpopulation. These results suggest that LDs might be used as a functional marker for CR-CSC identification and that Raman microspectroscopy holds a great potential for translational research on cancer stem cells. Raman microspectroscopy [191] is indeed a label-free technique based on vibrational spectroscopy; that is, imaging of samples is performed by probing, with subcellular resolution, and by optical means and molecular vibrations, which are specific to chemical bonds and structures of the molecules. This imaging method, which uses sample chemical composition as a contrast mechanism, is particularly suited to probe lipid biomolecules, which has been recently exploited to uniquely obtain quantitative chemical information on LDs (e.g., lipid saturation degree and cholesterol content) in live cells [178, 192].

A similar correlation between LDs, CD133, and Wnt/*β*-catenin has been proved in human metastatic melanoma cells, where the downregulation of CD133 resulted in reduced Wnt/*β*-catenin pathway signaling and decreased levels of LDs, as observed by Raman microspectroscopy, with a consequent reduced metastatic potential [193, 194].

Altogether, these findings corroborate the idea that LD accumulation and profile could have an important role on tumorigenic properties of CSCs and could represent a potential novel target for cancer prevention or treatment options.

## 8. Conclusions and Future Perspectives

Metabolic reprogramming in tumor cells is now considered one of the hallmarks of cancer [84], and it includes not only the increase of glucose uptake resulting in favoring glycolysis but also the upregulation of glutamine and lipid metabolism [195–197], aiming at sustaining rapid cell proliferation and biomass production. Increased de novo FA synthesis is a feature of many cancer cells and results in increased accumulation of LDs [115].

Different classes of drugs have been demonstrated to inhibit diverse lipid pathways, both in a direct and indirect way although no specific LD inhibitors have been described so far; examples of them are nonsteroidal anti-inflammatory drugs [198, 199] and statins [200]. Even if the mechanism of action of these drugs is not yet completely understood, they have exhibited promising results in the prevention of the CR cancer, suggesting a pivotal role for the LDs in CR-CSCs. Moreover, very recent results are indicating an even tighter connection between lipid metabolism and stemness [131, 178, 201, 202].

While it is becoming clear that LDs are involved in multiple cellular processes, their role in cancer and cancer stem cells needs further investigations. Research has made remarkable progresses in this field, but many questions regarding the LD biology still remain unanswered. In this regard, the questions arise as to whether differences in LD composition between healthy and cancer cells and among different tumors exists. Moreover, which functional roles have LDs in tumorigenesis and which advantages do they confer to CSCs in terms of tumorigenicity? Whether, throughout tumor development, CSC pool maintains itself uniform and stable or different CSC subclones originate with different stemness features at different stages is another open question. Accordingly, do LD expression levels change dynamically during cancer progression and, if so, how the tumor microenvironment may influence this expression? Investigating the modulation of LD expression and understanding their functional role will be of pivotal importance also for developing new potential strategies and targets for diagnosis and therapeutic purposes. Interdisciplinary approaches bringing together techniques like mass spectrometry, nuclear magnetic resonance, and Raman spectroscopies will allow performing new and deeper investigations of lipid phenotype, fatty acid composition, and spatial distribution of lipids and LDs inside tumors. Noticeably, Raman microspectroscopy adds to this set of techniques its unique capability to perform chemical imaging with high spatial resolution in live cells and without addition of any exogenous tag, as such being suitable even for *in vivo* applications. Moreover, it has been recently benefited by a quantum leap in technical development, through so-called “coherent Raman” methods, which have paved the way to quantitative investigations of lipid dysregulation in live cancer cells with spatial and temporal details inaccessible to other methods [203].
